# Applying the NHS restorative dentistry index of treatment need to clinical decision-making in Saudi Arabia

**DOI:** 10.1016/j.sdentj.2024.11.014

**Published:** 2024-12-02

**Authors:** Raghad A. Al-Dabbagh, Abdulelah J. Alrasheedy, Mohammed K. Jawi, Afaf A. Almabadi, Osama M. Felemban

**Affiliations:** a Oral and Maxillofacial Prosthodontics Department, King Abdulaziz University Faculty of Dentistry, Jeddah, KSA; b King Fahad Armed Forces Hospital, Jeddah, KSA; c Rama Medical Group, Jeddah, KSA; d Pediatric Dentistry Department, King Abdulaziz University Faculty of Dentistry, Jeddah, KSA

**Keywords:** Decision-making, NHS restorative dentistry index of treatment need, Saudi Arabia

## Abstract

**Objective:**

Making appropriate referrals for patients with complex restorative needs can be challenging. The NHS Restorative Dentistry Index of Treatment Need (IOTN) is an important guideline and tool that helps to ensure that patients receive the appropriate level of restorative dental care for their needs. The objective of this study was to test, via structured training, the usability and utility of the IOTN as a screening tool for referral and management of patients attending [King Abdulaziz University Faculty of Dentistry (KAUFD)], Saudi Arabia.

**Methods:**

In this prospective, interventional study, one hundred and fourteen participants (38 sixth-year dental students, 44 interns, and 32 general dentists) were equally and randomly assigned to either receive training on the IOTN or no training. Then, participants were provided with clinical and radiographic records for six cases and asked to make management and referral decisions recorded on a questionnaire.

**Results:**

The group receiving training on the IOTN more accurately classified cases into different levels of complexity and specialty and more frequently made correct management and referral decisions for complexity levels 2 and 3 cases than the untrained group. There was no difference between the trained and untrained groups for complexity level 1 cases.

**Conclusion:**

Training general dentists to use the NHS IOTN improved referral and management decision skills.

## 1 Introduction

Referral guidelines are evidence-based recommendations and protocols that help healthcare professionals determine when and how to refer their patients to specialists or consultants for specialized care (American Dental Association Council on Dental Practice, 1989, Falcon et al., 2001, Dawood and Patel, 2017, British Society of Periodontology and Implant Dentistry, 2020, Essam et al., 2021, Almohaimede et al., 2022, Porwal et al., 2022, Wei et al., 2022). In dentistry, these guidelines are designed to ensure that patients receive the most appropriate and effective dental care, whilst also promoting efficient use of resources and reducing unnecessary referrals. Typically, these guidelines include information on the types of cases that should be referred to specialists, as well as criteria for referral. These criteria may include factors such as case complexity, the patient's medical history, and the expertise of the referring dentist. Guidelines may also include information on the types of specialists that should be consulted for different types of cases, such as periodontists, endodontists, or orthodontists (Falcon et al., 2001, American Dental Association, 2007, Counihan et al., 2013, Dawood and Patel, 2017, British Society of Periodontology and Implant Dentistry, 2020, Almohaimede et al., 2022, Porwal et al., 2022, Wei et al., 2022). Making appropriate referrals for patients with complex restorative needs can sometimes be challenging, especially for clinicians with less experience, but by taking advantage of resources like referral guidelines and consultation networks, clinicians − provided that they are aware of their limitations and refer patients to specialists when needed − can ensure that their patients receive the best possible care to produce the best treatment outcomes (Falcon et al., 2001, Dawood and Patel, 2017, British Society of Periodontology and Implant Dentistry, 2020, Almohaimede et al., 2022, Porwal et al., 2022, Wei et al., 2022).

Specific guidelines may vary depending on the country, region, or even the individual dentist, and various dental screening tools have been proposed that classify patients according to their dental treatment needs and guide on appropriate referrals (Falcon et al., 2001, J., R. S. F. a. F., 2006, Muthukrishnan et al., 2007, Glickman, 2009, Saif et al., 2011, Seoane et al., 2011, Kakudate et al., 2015, Maheswari et al., 2015, Tonetti et al., 2018, British Society of Periodontology and Implant Dentistry, 2020, Ndjidda Bakari et al., 2021, Almohaimede et al., 2022, Cheng et al., 2022, Porwal et al., 2022, Wei et al., 2022) The United Kingdom National Health Service (NHS) Restorative Dentistry Index of Treatment Need (IOTN) is a tool that considers a range of factors to assess the extent and complexity of restorative dental treatment needed by a patient (Falcon et al., 2001, Muthukrishnan et al., 2007). Restorative dentistry involves the study, examination, and treatment of diseases of the oral cavity, teeth, and supporting structures, and it has three subspecialties: periodontics, endodontics, and prosthodontics. The IOTN index was introduced by the UK NHS to ensure that patients receive appropriate and consistent levels of restorative care. The IOTN aims to provide a standardized approach to restorative dental care that is fair, clear, and effective and ensures that patients receive the most appropriate treatment for their needs (Falcon et al., 2001). With the IOTN, once the dental examination is complete, the dentist assesses the patient's treatment needs and determines which NHS dental treatment complexity level the patient falls under. Treatment complexity ranges from complexity 1, which covers basic preventive care such as check-ups and simple treatments like fillings, scaling, and uncomplicated root canal treatment, to complexity 3, which covers more complex and costly treatments such as complex crowns, highly difficult endodontic treatments, and periodontic surgery. The IOTN helps to ensure that patients receive the appropriate level of care for their needs. One of the key benefits of the IOTN is that it helps to ensure that patients are not over- or under-treated. By categorizing patients into different case complexities based on their oral health needs, dentists can provide personalized care tailored to each patient's unique situation, thereby improving the overall quality of care provided to patients and ensuring that resources are used efficiently (Falcon et al., 2001).

Overall, the IOTN is an important tool in modern dentistry that helps to ensure that patients receive the appropriate level of restorative dental care for their needs while promoting transparency and fairness in the provision of dental services. However, there are no data on the transferability and useability of the IOTN for referring patients in the Saudi dental system. Thus, the aim of this study was to evaluate the need for training on the IOTN prior to possible future implementation in Saudi Arabia. Accordingly, the null hypothesis was that training general dentists in the IOTN has no effect on their referral and management decision skills.

## 2 Methods

### 2.1 Study design

In this prospective, interventional study, a convenience sample of 114 participants [n = 38 sixth year dental students, n = 44 interns, and n = 32 general dentists ([KAUFD] graduates)] were randomly divided into two groups (n = 57 per group) using the online Research Randomizer tool (https://www.randomizer.org): (i) a trained group, who received training on the IOTN, and (ii) an untrained group, who did not. Participation was optional, and accepting to answer the questionnaire was considered as providing consent. Answers were anonymous, and the Research Ethics Committee at KAUFD approved the study (approval number: 056-02-23).

### 2.2 NHS IOTN training

A lecture was delivered to participants in the trained group, which lasted 60–90 min and was delivered using a PowerPoint presentation. The lecture content included: (i) a description of the NHS IOTN; (ii) a demonstration of how to use the index by walking through three cases, each representing a different level of complexity; (iii) after ensuring that the audience understood the index, they were provided with three additional cases, one at each level of complexity, and asked to solve them on their own to verify that they understood how to properly utilize the IOTN. Untrained participants were not exposed to this lecture nor training.

### 2.3 Case assessment

All participants were provided with relevant clinical and radiographic records/data of six cases (two cases per IOTN complexity level). The cases were selected as being diagnosable from the radiographic and photographic records. They were then presented to consultants in periodontics, endodontics, and prosthodontics to confirm that the records were sufficiently clear for a diagnosis in each field. This process ensured accurate diagnoses, and the consultants confirmed the diagnosis and treatment plan. In addition, participants were provided with an electronic questionnaire in PDF format that included questions about participant sex, years of experience (answered by GPs only), level of case complexity, the highest discipline in complexity, and the treatment option (whether to treat the whole case, just part of it, or to refer the case) and, in cases of referral, which department the case would be referred to (Table 1). The untrained group was provided with relevant clinical and radiographic records/data of the same six cases and a similar questionnaire with one modification, i.e., they were not asked about the IOTN case complexity and management.

**Table 1:** Summary of the included cases.

Case number	NHS complexity	Highest discipline in complexity	Management	Referral
Case 1	1	Periodontics; needed scaling Fixed prosthodontics; needed restorations	General practitioner could provide treatment	Not needed
Case 2	1	Periodontics: needed scaling Fixed prosthodontics; needed restorations	General practitioner could provide treatment	Not needed
Case 3	2	Fixed prosthodontics; needed crowns	Based on the skill of the practitioner, could provide treatment	Based on the skill of the practitioner, could refer
Case 4	2	Fixed prosthodontics; needed fixed partial denture	Based on the skill of the practitioner, could provide treatment	Based on the skill of the practitioner, could refer
Case 5	3	Periodontics; needed implants Fixed prosthodontics: needed implant-supported prosthesis and extracoronal restorations that required anterior guidance modification	General practitioner could provide part of the treatment	Refer to periodontist and prosthodontist
Case 6	3	Removable prosthodontics; needed surveyed crowns	General practitioner could provide part of the treatment	Refer to prosthodontist

Two cases of each NHS index complexity were included in the assessment of referral skills. Cases were presented in a random order to each participant. Cases 1 and 2 were complexity 1 cases, and both cases required simple periodontal care such as scaling and oral hygiene reinforcement and caries management, where the proper management was to complete the treatment without any referral. Cases 3 and 4 were complexity 2 cases due to the need for crowns and fixed partial dentures, respectively. In these cases, the clinician had the choice to refer the case or to complete it themselves, depending on their clinical skills and ability to perform the procedures competitively. Cases 5 and 6 were complexity 3 cases that required referral to a specialist/consultant due to the difficulty in management. Case number 5 needed implants and an implant-supported prosthesis and had a deep bite problem which were considered complexity 3 for both periodontics and fixed prosthodontics disciplines. According to the index, the case needed to be handled by a periodontist/prosthodontist to finalize the treatment. Case number 6 needed surveyed crowns and a removable partial denture and required referral to a prosthodontist after caries control and scaling (which could be provided by the primary care clinic/GP). Case numbers 4, 5, and 6 also needed simple surgical intervention or minor surgery (extractions), and those procedures were not mentioned in the index but could be managed by the general practitioner or referred to a specialist depending on ability.

### 2.4 Data analysis

Descriptive and inferential statistics were used to describe the demographic data and to compare the effects of IOTN training on the referral skills of participants, respectively. Differences in age between groups were compared with the independent sample *t-*test. Categorical data were compared between groups using the chi-squared test. A *P*-value < 0.05 was considered significant. IBM SPSS Statistics for Windows, v29.0.2.0 (IBM Statistics, Armonk, NY) was used for data analysis.

## 3 Results

The demographic characteristics of the study participants are presented in Table 2. There were no significant differences in age (*P* = 0.503), sex (male to female ratio 2:1; *P* = 0.841), nor stage of training (*P* = 1.00) between the trained and untrained groups. For general dentists, the majority had less than five years of experience, with a similar distribution between the two groups (*P* = 0.595). Most general dentists had less than five years of experience due to the convenience sampling approach used. As a result, it was challenging to find a larger number of experienced general dentists with a similar distribution between the groups.

**Table 2:** Demographic characteristics of the study participants.

		**IOTN trained** **n = 57 (%)**	**IOTN untrained** **n = 57 (%)**	***P*-value**
Age	Mean ± SD	25.2 ± 1.6	25.5 ± 2.7	0.503†
Sex	Male	39 (68.4)	38 (66.7)	0.841€
	Female	18 (31.6)	19 (33.3)	
Group	6th year student	19 (33.3)	19 (33.3)	1.00€
	Intern	22 (38.6)	22 (38.6)	
	General practitioner	16 (28.1)	16 (28.1)	
Years of experience (general dentists only)	Less than 5 years	14 (87.5)	13 (81.3)	0.595€
	6–10 years	2 (12.5)	2 (12.5)	
	11–15 years	0	1 (6.3)	

† Independent sample *t*-test.

€ Chi-squared test.

**Fig. 1:**
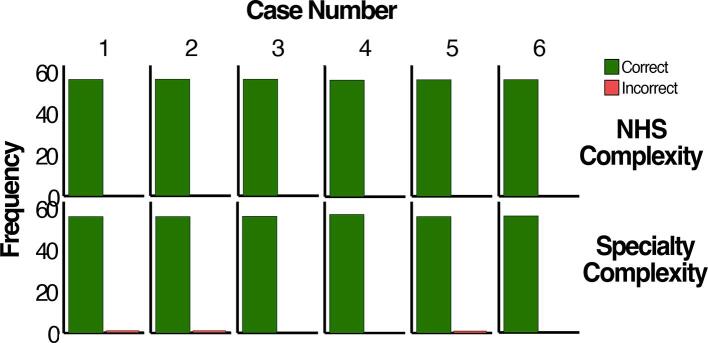
Trained participants' ability to correctly identify a case complexity and referral according to the NHS IOTN.

Trained participants were able to correctly classify cases into different levels of complexity and specialty (Fig. 1): all participants correctly classified the complexity of the six cases, and most correctly identified the specialty.

We next examined the effect of IOTN training on participants' decisions to manage and refer, where participants could decide to treat, refer, or both to one of six specialties: periodontics, restorative dentistry, endodontics, prosthodontics, orthodontics, and oral surgery. In general, training had a significant positive effect on management (*P* < 0.001) and referral (*P* < 0.001) decisions, i.e., whether to treat or refer, in cases of higher complexity. NHS IOTN training improved the decision-making process of participants to make correct management (rate ratio (RR) = 1.3) and referral decisions (RR = 1.3) by 30 % compared with the untrained group (Table 3).

**Table 3:** Overall effect of NHS IOTN training on management and referral decisions.

		Trained n = 342	Untrained n = 342	*Chi-squared value*	*df*	*Rate Ratio* *95 % CI*	*P*-value
Management decision	Correct	239 (69.9)	189 (55.3)	15.6	1	1.3 (1.1 – 1.4)	<0.001€
	Incorrect	103 (30.1)	153 (44.7)				
Referral decision	Correct	175 (51.2)	131 (38.4)	11.2	1	1.3 (1.1 – 1.6)	<0.001
	Incorrect	167 (48.8)	210 (61.6)				

Abbreviations: df − degrees of freedom, CI − confidence interval.

€ Chi-squared test.

**Fig. 2:**
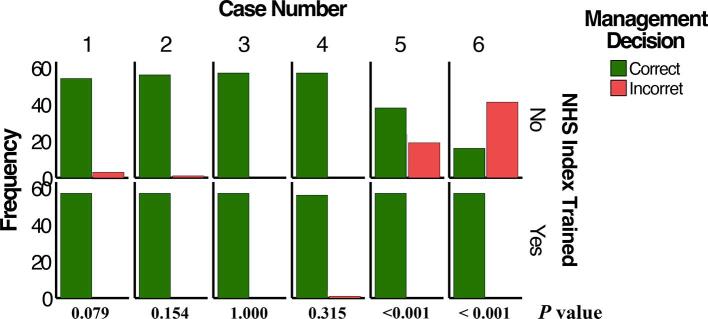

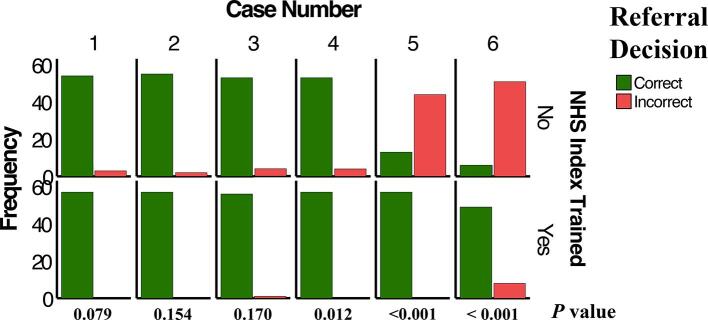
(A) Comparison of trained and untrained participants with respect to identifying the correct management of the six assessed cases according to the NHS IOTN. (B) Comparison of trained and untrained participants in identifying the correct referral for the six assessed cases according to the NHS IOTN.

The effect of IOTN training on case-level management (Fig. 2A) and referral (Fig. 2B) decisions were next evaluated. For complexity 1 cases (cases 1 and 2), there were no significant differences in decision-making between trained and untrained participants for both management and referral decisions (*P* > 0.05). For one complexity 2 case (case 3), there was no difference in management nor referral decisions between trained and untrained groups (*P* > 0.05). In the other complexity 2 case (case 4), while there was no difference between groups in terms of management decision (*P* = 0.315), there was a significant difference in decision to refer (*P* = 0.012), with trained participants having a significantly higher rate of correct decisions (100 %) than those in the untrained group (89.47 %). This case required fixed partial dentures, for which the general practitioner could choose to refer the case or manage it themselves, depending on their clinical skills and ability to perform the procedures competently. Most participants in both groups chose to treat the case, with a significantly higher rate in the trained group (82.5 %) than the untrained group (64.9 %) (*P* = 0.042). The specialty of choice for referral was prosthodontics (17.5 % for trained group and 26.3 % for the untrained group).

For complexity 3 cases (cases 5 and 6), the trained group had a significantly higher rate of correct decisions for both management and referral compared with the untrained group (*P* < 0.001), with the trained group making the correct decisions 100 % of the time and the untrained group making the correct management and referral decisions 28.1 % and 8.78 % of the time for case 5 and 66.7 % and 22.8 % of the time for case 6, respectively. Case 5 required referral to a specialist/consultant periodontist and a prosthodontist due to the need for implants, implant-supported prosthesis, and a deep bite problem. All participants in the trained group chose to refer the case, while most participants in the untrained group chose to treat part of the case then refer (*P* < 0.001). The most frequent referral specialties for the trained and untrained groups were periodontics (100 % and 77.2 %), restorative dentistry (86.0 % and 17.5 %), prosthodontics (100 % and 75.4 %), orthodontics (96.5 % and 54.4 %), and oral surgery (86.0 % and 17.5 %), with a significant difference observed between trained and untrained participants regarding referral specialties (*P* < 0.001).

Case 6 required referral to a prosthodontist after caries control and scaling that could be provided by the GP due to the need for surveyed crowns and an RPD. All trained participants chose to treat then refer the patient to a prosthodontist for surveyed crowns and an RPD or to an orthodontist to correct the deep bite (1 out of 57 participants) or oral surgeon (1 out of 57 participants). A significant proportion of participants in the untrained group chose to treat the case (31.6 %), with the majority choosing to treat and refer (*P* < 0.001). Referrals to different specialties were significantly different between the two groups for endodontics and prosthodontics (*P* < 0.001), with all trained participants choosing referral to prosthodontics (100 %).

Cases 4, 5, and 6 needed simple surgical intervention or minor surgery (extractions), and while those procedures were not mentioned in the index, they could be managed by the general practitioner or referred to a specialist depending on ability.

## 4 Discussion

Restorative dentistry is indicated for complex dental problems that require coordinated management from several dental specialties. These problems range from mild (e.g., restorative treatment for damaged teeth) to severe conditions requiring full mouth rehabilitation. The NHS IOTN includes three main specialties − periodontics, endodontics, and prosthodontics − each with a series of codes that eventually classify a case into one of three complexity levels. For professional use of the index, each component should be assessed separately, and the overall restorative complexity of the case expressed as the highest component code (Falcon et al., 2001). During complete assessment of the case, modifying factors such as underlying medical conditions may be present that must also be considered, as they can increase the complexity score (Falcon et al., 2001). By using the index correctly, clinicians can ensure that patients receive the most appropriate care, thereby improving outcomes and reducing healthcare costs. To our best knowledge, this is the first examination of the NHS-RID in treatment decision-making in another country or Saudi Arabia.

Here we investigated the impact of IOTN training on clinicians' ability to make appropriate management and referral decisions for patients with complex restorative needs. Our results show that training may have a positive impact on decision-making, as evidenced by the significantly higher rates of correct decisions made by the trained group in many cases. Additionally, the trained group had a high rate of correct complexity classification, complexity specialty decisions, referral decisions, and management decisions. This finding is important, as accurate classification of cases is crucial for ensuring that patients receive the most appropriate and effective care. Receiving training in and using the NHS IOTN may help clinicians to accurately classify the complexity of cases and identify the most appropriate management. The NHS IOTN was also clear, reliable, useful, and did not take a long time to understand, i.e., it is a user-friendly tool that can be easily integrated into clinical practice.

Referral guidelines vary according to geography. There are currently no consensus dental referral guidelines in Saudi Arabia, with each institution having its own referral system. For example, dental referrals are easily performed through the electronic medical records system (R4), with students and general dentists able to refer patients to the intended department according to patient need. For students, referrals must be approved by specialists and consultant clinical supervisors. Nevertheless, a restorative assessment and referral guideline is still needed to optimize referrals, not only to meet patients’ needs and desires but also to standardize the process to ensure equity in dental service provision and to help decision-making, especially for junior dental staff.

Restorative dentistry aims to preserve the remaining tooth structure and restore the health of the teeth and supporting oral structures, thereby restoring function, aesthetics, and comfort. This often requires referral to specialist services (General Dental Council 2022). According to the Saudi Commission for Health Specialties, doctors are responsible for referring patients to other, specialized doctors when required, without delay and with all relevant documentation (Commission for Health Specialties, 2014). This need has led to fast electronic referral systems but, nevertheless, poor referral information may lead to inefficient management (Morris and Burke 2001). In Saudi Arabia, the Ministry of Health has implemented a unique electronic referral system (Ehalati e-referral system) to refer patients from Primary Health Care (PHC) clinics to specialized hospitals in an efficient and practical way (Ministry of Health 2004). Indeed, dental referrals have been shown to be one of the most frequent referrals on the system, but some are improperly made, with some patients inappropriately treated in PHCs rather than being referred. This is at least in part due to an absence of dental referral guidelines (Alabbasi et al., 2022). To overcome this problem, the UK set up Managed Clinical Networks (MCNs) to integrate services and allow assessment of the quality and suitability of referrals (Sivarajasingam et al., 2020). Incorporating such a model within the electronic dental referral software in Saudi Arabia to facilitate referral tracking and determine inappropriate referrals would be an innovative development that might help to improve service efficiency. In a similar initiative, the Saudi Medical Appointments and Referral Centre (SMARC) was introduced in 2019 at the MoH to coordinate and approve emergency medical referrals, which has since been shown to facilitate proper referrals and reduce mortality rates. In this system, each medical emergency referral is sent to the Office of Coordination and Eligibility for Treatment (OCET) for approval (Aljerian and Alharbi 2024).

In general dentistry, the general dentist delivers treatments approved by the regulatory body according to individual competencies achieved during undergraduate studies. Therefore, in Saudi Arabia, general dentists working in PHCs must perform certain tasks including patient examination, diagnosis, and treatment planning as well as simple pedodontics, periodontics, endodontics, and restorative treatments together with simple extractions, emergency dental infection management, and preventive treatments (Ministry of Health 2004). By contrast, general dentists in the UK can provide extended dental services such as replacing missing teeth, restoring damaged teeth, and providing periodontal and root canal treatment. The Saudi MoH classifies dental service provision into three levels: (1) primary dental care practices that serve the community within the area of the practice through caries control with dental fillings, extractions, emergency, and preventive treatments; (2) dental clinics in specialty hospital services, which provide minor oral surgery and fixed and removable prosthodontics; and (3) dental clinics in consulting hospitals, which serve patients in all dental specialties and provide dental training (Ministry of Health, 2004, Almohaimede et al., 2022). Nevertheless, there are no MoH referral guidelines, which may result in confusion in decision-making, especially for new dental graduates. To address this, and by using the NHS-RID correctly, clinicians can ensure that patients receive the most appropriate care from the most appropriate specialists, thereby improving treatment outcomes and reducing healthcare costs.

We found that the complexity of the case had a significant impact on participants' decision-making abilities, particularly for more complex cases (complexity 2 and 3). This impact was seen for both referral and management decisions, and training on the IOTN altered decision-making in some cases. While complexity 3 cases require specialist referral, level 2 complexity can either be treated by general dentists or referred, depending on the case. Participants made correct management and referral decisions for all complexity 1 and some complexity 2 cases, which fall within the competency level of general dental practitioners. However, in three cases, there were significant differences in both management and referral decision-making between the trained and untrained participants, indicating that training and using the IOTN had a positive impact on appropriate and accurate decision-making for the most complex cases. This finding is consistent with a previous report from Saudi Arabia, where there was agreement between dental interns and new graduates in restorability assessment and tooth prognosis for simple cases of mild complexity but not for more complex cases (complexity level 2) (Aldowah 2022). This can be attributed to their confidence in treating simple cases commensurate with their level of experience. However, evidence in this area is very limited, since there is no widespread adoption of the IOTN by dental students and graduates.

Decision making is an indispensable skill in dentistry that is guided by balancing confidence and competency. Appropriate decision-making leads to avoidance of over- or under-treatment and can ideally be achieved by following structured frameworks (indices) (Hamer et al., 2021), such as by training dental students in the use of the IOTN to significantly improve their decision agreement (Bentele et al., 2002). The findings also highlight the importance of ongoing training and support for healthcare professionals to ensure that they make appropriate and accurate decisions in the best interests of their patients, consistent with the findings of other studies where dental training improved the confidence level and treatment planning decisions of dental students (Hamer et al., 2021). This is especially true for general dentists working in developing countries, who encounter more complex restorative cases due to the high incidence of caries, including in Saudi Arabia (Alshammari et al., 2021).

For case 5, untrained participants choosing incorrect referrals only referred the case to a periodontist and prosthodontist, while this patient in fact needed referral to a periodontist for implant placement, a restorative dentist for restorations, and a prosthodontist to restore the implant-supported crowns. In this case, there were significant differences between the two groups in terms of referrals to all departments, which may reflect an ambiguous clinical scenario where standardization of dental care using the restorative index as a reference is necessary. If participants made any of these referrals, the decision was considered correct. Such complex cases might be difficult for general dentists lacking clinical experience due to limited exposure to diverse clinical scenarios, a problem that might be magnified as learning outcomes vary from the different Saudi universities (Aldowah 2022).

Our findings show that the IOTN can help to reduce variation in practice across different regions and healthcare settings through all dental healthcare providers using the same tool to assess the complexity of cases and make treatment decisions. However, the application of this index in Saudi Arabia may be challenging, due to geographic and cultural differences. In the UK, public dental health programs and public awareness about dental health are well-established and more advanced than in Saudi Arabia (Al-Hazzaa et al., 2014). Early childhood dental disease prevention programs are widespread and focus on early disease prevention and intervention. Practitioners in Saudi Arabia may face more complex dental cases due to the high prevalence of caries, neglect of oral hygiene, and poor access to dental healthcare for routine checkups, where pain is often the main driver to seek dental treatment (Alayadi et al., 2019, Aqeeli et al., 2021); this provides further motivation to apply the IOTN to unify clinical decision-making and reduce personal subjectivity. The presence of dental care barriers (distance to dental care centers, dental anxiety, population awareness, long waiting lists, limited oral health promotion, and socioeconomic level) may be limiting factors to the efficient use of the index for dental referrals (Al Agili and Farsi, 2020, Almajed et al., 2024). Furthermore, other factors may influence implementation of the index in practice, such as challenges related to technology integration and use, such as through staff resistance, poor technical skills, and usability-related issues (Almaiman et al., 2014). Supporting dental training and diverse student experiences in dentistry in Saudi universities also has financial implications, and there are differences in learning outcomes between accredited and well-established universities and newly developing dental colleges (Aldowah 2022). Other relevant factors include cultural and social differences in Saudi populations regarding oral health, perception of esthetics, and socioeconomic status, all of which can affect the delivery and acceptance of dental management (Almajed et al., 2024), and the government and private dental sectors do not always agree on clinical treatment decision-making and patient preferences (Espelid et al., 2001, Vidnes-Kopperud et al., 2011, Alshahrani and Raheel, 2016, Suliman et al., 2020). Despite these barriers, the clinical benefits of applying the index can outweigh the limiting factors.

The study has some limitations, including its single center design and the inclusion of dental students, interns, and general practitioners with less than five years of experience. Therefore, the results may not be generalizable to more experienced practitioners or those at different stages of their careers. However, senior undergraduates, interns, and general dentists were the sample of interest, as their referrals are mostly for more specialized dental care. Additionally, we did not assess the long-term impact of IOTN training on decision-making nor patient outcomes, which are important considerations for evaluating the effectiveness of training. Although we carefully selected a range of clinical scenarios, clinical presentations may differ in practice and be more challenging. We did not assess the retention period of knowledge about index training, which might indicate the need for reinforcement training. Despite these limitations, we minimized potential confounding variables, as the groups were matched for age, sex, group, and years of experience.

Given our findings, training in the IOTN may be valuable to facilitate accurate decision-making, and wide clinical implementation and training, as outlined here, could be considered as part of the core implementation strategy for referral guidelines. To quality-assure dental training and to facilitate this, the IOTN could be implemented into the clinical training of senior year undergraduate dental students. Accordingly, multi-center studies where training is embedded in the curriculum are now needed to explore the use of the IOTN for screening patients for restorative dentistry needs in Saudi Arabia and its impact on clinical and health economic outcomes.

## 5 Conclusions

The correct identification of the complexity of different restorative cases is a crucial pre-requisite for an appropriate and proficient referral. The results of our study suggest that training general dentists with NHS IOTN could improve referral and management decision skills.

## Ethical statement

The research was conducted in accordance with the principles embodied in the Declaration of Helsinki and in accordance with local statutory requirements. All participants gave informed consent to participate in the study.
